# Genomic Data Sharing: Navigating The Ethical Issues In Collaborative Research Relationships Between The Global North-South

**DOI:** 10.58177/ajb240007

**Published:** 2024-09-06

**Authors:** Deborah Ekusai Sebatta, David Kyaddondo, John Barugahare, David Kaawa-Mafigiri, Shenuka Singh, Erisa Mwaka

**Affiliations:** 1. Makerere University, College of Health Sciences, Department of Anatomy, Kampala, Uganda; 2. Makerere University, College of Health Sciences, Department of Child Health and Development Centre, Kampala, Uganda; 3. Makerere University, College of Humanities and Social Sciences, School of Liberal and Performing Arts, Department of Philosophy, Kampala, Uganda; 4. Makerere University, College of Humanities and Social Sciences, Department of Social work and Social Administration, Kampala, Uganda; 5. University of KwaZulu Natal, Discipline of Dentistry, Durban, South Africa

**Keywords:** Data sharing, genomics, equity, trust, fairness

## Abstract

**Background:**

In recent years, Africa has seen a significant growth in genomic research, leading to the sharing of participant data between researchers in Africa and across the globe.

The study aimed to investigate the ethical issues that impacted the sharing of genomic data during collaborative research and explored strategies to promote equity.

**Methods:**

This was a phenomenological qualitative study that utilized the told experiences of key informant interviews to collect data from August 2023 to March 2024. A total of 38 key informants participated in this study, of whom 28.9% were female, and their ages ranged between 30–64 years. The participants included researchers involved in genomics research (16), members of research ethics committees (14), and officials from national regulatory bodies. (8) Interviews were transcribed verbatim and analyzed using thematic analysis and NVivo software 14 was utilized to assist in data management. A total of four main themes emerged: (mis)trust in collaborative research, inequitable access to data due, power differentials, and data sharing as a form of ethical imperialism.

**Results:**

The study revealed (mis)trust in collaborative research was influenced by fear of data misuse, and no framework for benefit sharing. (In)equitable access to data by researchers from low and middle-income countries (LMICs) was attributed to poor infrastructure and limited skill. Researchers in LMICs could be compromised because of the power differentials with their counterparts from the North, resulting in exploitation. Data sharing could be perceived as a form of ethical imperialism by the North imposing its culture on the South.

**Conclusion:**

Establishing relationships with international collaborators that are built on trust and equity is critical. Policymakers should consider developing regulatory frameworks that foster equity and fairness in data sharing and access in collaborative research. This will be facilitated by engaging in active advocacy dialogues with funders, and policymakers, and researchers from both the North and South. Researchers from the Global South should have their capacities strengthened and empowered so they have the skills necessary to access and utilize the data as well as advocate favourably.

## Introduction

Advancements in technologies like gene sequencing have made genomics research more frequent (1, 2). An increasing number of government agencies, funding organizations, and publishers advocate for more data sharing (3). In recent years, Africa has also seen significant growth in international collaborative genomic research, drawn into the sharing of participant data between researchers in Africa and across the globe for reasons such as lack of the needed technological capacity to analyse the data and donor requirements.

Data sharing has played a crucial role in the progress of genomics research in Africa and worldwide. Genomics research heavily relies on the sharing of data via global collaborative networks (4, 5). The primary objective of the funders is to make data more accessible to all researchers across the globe (3).

While data sharing can be an efficient and effective way to lead to scientific and potential public health benefits, several ethical, legal, and societal issues (ELSI) are associated with sharing genomic data in collaborative research. Some of the ELSI include the type of informed consent to be used during data sharing, breach of participant privacy and confidentiality, data breaches, the fear of misuse of data on the side of the Northern collaborators and justice and (in)equity in research (6, 7).

International research collaborations have accelerated efforts to promote Global data sharing through the Global Alliance for Genomics and Health (8). However, among the major challenges in forming partnerships with Northern collaborators is mistrust from previous data misuse as well as unequal power relations between partners (9). Usually, worries about inequities arise from unequal vantage points between researchers from the Global North and South about access to data, benefit sharing, limited opportunities to publish scientific articles due to time and structural constraints such as data ownership, power dynamics, knowledge, capacities, and capabilities (10). Low- and Middle-Income Countries (LMICs) face several challenges, including low digital literacy, inadequate infrastructure, and limited investment in data science training. These challenges make it difficult for LMICs to access and utilize the data (3).

The Global divide between North and South is primarily based on the level of socioeconomic development, where the North consists of high-income countries and the South comprises low-income countries (11). The difference between the North-South partnerships is because the funding comes from the North and with conditions like unlimed acess to data by third party companies and it is from here that inequalities arise. In partnerships, differences may arise due to distinct assumptions, world views, agendas, and expectations. Although such differences can be seen in both North-North and South-South partnerships, they are more pronounced in partnerships between North and South or between South and North (12).

Many researchers in LMICS have a negative attitude to data sharing chiefly due to mistrust of collaborators from the global North, and the historical exploitation and inequity in Global health research (13, 14). However, there is a push towards open data sharing and several journals and funders of genetics and genomic research request for mandatory submission of datasets to open access repositories for the benefit of science. However, studies from Africa show that researchers are hesitant to share genomic data for various reasons (15, 16). Mistrust and inequity as a result of unequal power differentials have been identified as gaps in collaborative genomic research. The research undertaken has contributed significantly to raising awareness and generating additional evidence regarding the mistrust in collaborative genomic research.

This evidence should be leveraged to explore ways to promote equitable partnerships and prevent exploitation in collaborative genomic research.

The study aimed to investigate the ethical issues that impacted the sharing of genomic data during collaborative research and explored strategies to promote equity.

## Methods

### Study design

We used a phenomenological qualitative research design (17) that utilized told experiences of researchers, research ethics committee members, and those of regulators through Key informant interviews (KIIs) regarding sharing genomics data.

In this context, equity refers to access to shared data and the potential benefits derived from it.

### Setting

We conducted this study at Makerere University College of Health Sciences (MakCHS) and its affiliate research institutions located at Mulago National Referral Hospital Kampala, Uganda. MakCHS is one of the nine constituent colleges of Makerere University. The research affiliate institutions included were: Infectious Diseases Institute (IDI), Uganda Cancer Institute (UCI), Baylor Uganda, Makerere Biomedical Research Center (MAKBRC) and Infectious Diseases Research Collaboration (IDRC).

We also conducted research with National regulatory bodies namely: the Uganda National Council for Science and Technology (UNCST), National Drug Authority (NDA), Personal Data Protection Office (PDPO), Uganda National Health Research Organization (UNHRO) and the Ministry of Information and communication technology (ICT). Seven out of the 35 accredited RECs in Uganda were purposively selected because of their experience with reviewing genetic/genomic research (18). A total of seven RECs that review genomic research in Uganda namely: 1) School of Biomedical Sciences (SBS), 2) UCI, 3) UVRI, 4) Mulago Hospital REC (MHREC), 5) Lacor Hospital, 6) Joint clinical research centre (JCRC) and 7) National HIV/AIDS Research Committee (NARC) were involved in the study.

### Sampling

We used a purposive sampling technique and the maximum variation principle to select a diverse range of cases of studies on genomics to get variation among the researchers. Further, our criteria included selecting participants from different categories, such as those involved in the conduct of genomics research and those with experience in oversight. We aimed to capture their varying views on data sharing in collaborative genomic research (19, 20).

A list of MakCHS researchers who specialize in genomics research was compiled and they were invited to participate in the study. Further, a list of RECs that approve genomics research was downloaded from the UNCST website. The list was reviewed and participants were purposively selected to include: REC chairs, members and community representatives. The participants were contacted by phone or email respectively to take part in the study. Contact people in the national regulatory bodies were got in touch with and they helped identify key regulators with experience in research oversight and data protection. The respective members were then contacted to take part in the study.

### Participants

A total of 16 researchers were purposively selected out of 23 (18) who were either principal investigators or study coordinators of protocols related to host genomics and genetic research or with expertise in the law. We approached a total of 18 members of RECs who approve genomic protocols, of whom 14 agreed to participate and 4 declined who were mainly chairs and a few regular members. The 14 included REC chairs, members and community representatives. We also approached 8 regulators involved in research management and oversight, all of whom agreed to take part in the study.

A summary of the setting, sampling strategy, category of participants and sample size are summarized above (Table 1).

### Data collection

Between August 2023 and March 2024, a team of three researchers conducted 38 Key informant interviews (KIIs). The team consisted of the lead researcher, a research assistant and a note taker. Before starting the study, the research team underwent training on the study protocol, ethical issues in the conduct of research, a short orientation about genomics and on the tools to ensure they understood the study well. A pilot study was conducted with two participants from each category from some of the institutions above to ensure clarity and length of questions. The KII guides that explored views on data sharing in genomics research were developed by the lead researcher (DES) and underwent multiple rounds of review by the Co-investigators. The team used KII guides to collect data from different categories of participants. The guides consisted of open-ended questions and were piloted and revised before the full data collection process. Participants belonging to different categories were asked “What are your views on genomic data sharing in collaborative research?” What are your concerns regarding data sharing in collaborative genomic research”?” How can we ensure equity in collaborative genomic research? The interviews lasted 45–60 minutes and were conducted in English. Detailed notes were taken alongside audio recordings of the interviews.

Debriefing meetings were held by the research team at the end of each interview to ensure completeness and to also review preliminary perspectives that had arisen.

### Research team and reflexivity

Two team members (DES) and (DK) were involved in the consent process and facilitation of the KIIs independently, and their decisions were not influenced by the study. One team member (IA) participated in taking notes during the process independently. The participants were encouraged to express themselves freely and were assured that their identity would remain confidential. We were aware that during the interviews, especially with research stakeholders, we needed to remain neutral, put aside our own views, and listen from the perspective of the respondents (18).

During the study and reporting process, the researcher remained unbiased despite prior involvement in collaborative studies with researchers from the North regardless of the experiences and the assumptions.

### Data management and analysis

The audio recordings were transferred to password-protected computers and deleted from the recorders (21). All audio recordings were transcribed verbatim. The lead researcher verified the transcripts to ensure their accuracy and authenticity with respect to the audio recordings. All data was de-identified to maintain confidentiality and protect participant identity. The authors adopted a team-based approach to analyze the data, as described by Ekusai-Sebatta et al. (2021). This involved reading the transcripts to help the authors familiarize themselves with the data, mark important sections, and make notes (22). The team (DES, JB, AS) developed a draft coding framework based on the first set of interviews conducted, then developed the final coding framework. A thematic approach of analysis was used in identifying, analyzing and the interpretation of the data (23).

The transcripts were initially analyzed separately for three categories of participants: researchers, REC members, and regulators. This was done to gain a deeper understanding of each category’s perceptions about the topic, as described by Nabukenya et al. in 2023. The social scientists (JB and AS) performed the coding process. The team iteratively discussed and synthesized codes from independent readings. The quality control was done by DES. The team examined the themes for patterns until a consensus was reached on the final themes (24). The hierarchies of codes were then sorted into categories based on how the themes were related and linked (22). The data was managed using NVivo software (version 14), and the presentation of findings followed the Consolidated Criteria for Reporting Qualitative Research (COREQ) checklist (25).

## Results

### Demographics

A total of 38 participants took part in the study, with 28.9% female and an age range of 30–64. The REC representatives included chairs as well as community representatives and members. Regulators were selected from National Regulatory bodies (Table 2) below.

Thematic analysis identified four main themes from the KIIs. These are summarised in the table below (Table 3):

### Theme 1: (Mis)trust in collaborative research

Researchers highlighted several trust issues that influence the sharing of genomic research data. Some researchers expressed willingness to share data for altruistic reasons, as their contribution to the advancement of science. However, they acknowledged the existence of inequity in genomic research.

One researcher with a positive attitude towards data sharing noted:

**R:** Well, I think data should be shared. In medicine, there are fields where people didn’t train colleagues and that field is dead. That healthy competition and having more people doing something brings in new ideas. They (the funders) are saying it should be available to anyone! Of course, there is some imbalance because people in the developed countries are more emancipated or have seen better trainings (Researcher 16).

On the other hand, several respondents were hesitant to share their genomic data, and this was attributed to several reasons. Some of the reasons highlighted included unclear motives of counterparts from high-income countries (HICs), fear of misuse of the data, use of data for commercial purposes without the consent of all collaborating parties, and inequity in the sharing of the benefits.

Two respondents opined about the fear of misuse and loss of control:

How do you restrain the researchers from limiting their objectives to the questions that they have asked? It becomes a problem. How do we ensure that they don’t use this information for questions that are not asked, questions that are not yet asked? How do we ensure that we control the use of this data beyond the immediate? It becomes a big problem once the data goes out to the different partners (REC 04 Community representative).

So, I made a presentation to a group and they were all very happy. So, after like two weeks, one of the professors wrote to me and asked me whether I was willing to share the primary data because they wanted to reanalyze it and we can collaborate and submit it. I am like; to share primary data, no. I called the professor friend who told me they [would] rub off your name, put in their grant, they get the money and you will never see them again. And I said; well, I don’t know whether I will share the primary data, but I can share with you my analyzed data and I never heard from him again (Researcher 08).

### Perceptions held by the Global South researchers about the North

A summary of the findings indicates that the attitudes of researchers from the global South toward their counterparts in the North have a significant impact on their willingness to share genomic data. Those who trust their collaborators from the North and believe that they have not acted improperly are more willing to share their data, while those who view the partnerships as exploitative are less likely to share their data

One respondent who had negative perceptions about the intentions of the collaborators and use of data noted:

Who watches then, that they do what they said they would do with the data even when the signing has been done? (Regulator 03)

### Trust of researchers from the Global South by the South researchers

Findings from the KIIs indicate South-South collaborations, countries may face similar development challenges, which results in a shared understanding of priorities, and they are more likely to share genomics data openly. Collaborative research among South-South researchers often faces limitations in capacity, unfair competition, and issues with building a name and authorship. Additionally, the findings revealed that academic institutions and researchers within South-South collaborations also faced challenges of mistrust, driven by concerns that they might claim ownership over participants and data, as well as the fact that all parties are focused on advancing their careers.

One respondent noted that regardless of whether the researcher was from the Global South or North, competition is always evident in an academic environment thus:

We are in an academic environment that is very brutal, I want to get ahead of you, I want to get the promotion, especially in research setups between researchers in the Global South. So, those are some of the things that push a bit of these wrong tendencies (researcher 09).

### Theme 2: Inequitable access to shared genomic data

Findings from the different categories of respondents interviewed indicated unequal access to data, especially for researchers from LMICs. The majority of respondents complained about unequal access to shared data, especially by researchers from LMICs. The reasons given for this included limited infrastructure, power disparities, digitalization and advances in data science, affordability, and lack of awareness. Some respondents were worried about losing control of their data once it is shared.

There appeared to be varied views among respondents, with one indicating that inequitable access to data led to mistrust in collaborative research.

R: We do not have control once the data is sent out, especially to our people in the global North. If data is sent there, sometimes we do not have control reason being, that we have limited resources, yes, maybe limited capacity to track, but also, the bargaining power. Most of the time, we are only receiving, and so, some of these proposals come in with stringent. So, you may find at the time of negotiation, you may not have the bargaining power to say how much you can protect the locals or protect yourself as an investigator (Regulator 1).

### Theme 3: Power differentials between researchers and institutions in the Global North-South collaborations

Most participants opined that there are power disparities at various levels that impact equity and fairness in collaborative research. They felt that power differentials put researchers from LMICS in a vulnerable position and heightened the risk of exploitation. Furthermore, they posited that the risk of exploitation is accentuated by the lack of appropriate ethical and legal frameworks. For example, one researcher noted that the lack of regulatory frameworks compromises researchers from the Global South during benefit and data-sharing negotiations for fear of losing potential collaboration opportunities.

Compromise comes at many levels.; The institution may need funds to sustain it and its staff. I think that’s the biggest area of compromise, funding. So, when the discussions become more difficult and especially where there are other alternatives to go to, which are willing to, maybe not to put up strong resistance so, you start weighing the prospect of losing a certain amount of money and then maybe you may compromise on some of these things. Now, coupled with the fact that maybe you probably don’t appreciate what the end product is going to be like (Researcher 15).

But also, most of our researchers here, are very vulnerable. They have limited knowledge about data sharing, even in interpreting and even understanding the existing laws. Like recently, I have been supporting an organization to fill data sharing agreements, but the organization doesn’t know what is in Data Protection Act of Uganda. They don’t know what is in the E-Data Act. Like if you are sharing genomics data, most of that data is E. So, people don’t know those laws (Regulator 06).

One respondent noted that researchers could be the reason they are compromised because they are always on the receiving end.

We are ever receiving from the North, where is yours on the table? I have also been a PI for certain protocols, I write, when I read these protocols, you find that in the background, they show you some gaps and they try to answer those gaps, you may find that there are some other gaps that may not be answered. Why don’t you jump on that also and write? (REC 13).

### Theme 4: Data sharing as a form of ethical imperialism

Several respondents contended that research funders/sponsors often impose unfavorable conditions on researchers from the Global South, and they opined that this was a form of ethical imperialism. However, there were contrasting opinions on whether the requirement for mandatory genomic data sharing was a form of ethical imperialism or not. Some participants argued that it is not, but they acknowledged that different cultures have unique beliefs and norms that shape their views on genomics and data sharing. The findings also demonstrate that Africans and especially students are very excited to share their data thinking it will come with immediate collaboration.

One respondent who did not agree that data sharing was a form of ethical imperialism noted:

R: No, I don’t think so. Those are individuals who position themselves as if they are being colonized again. You have to be strong, this is your data but our PIs are here in Uganda are not very strong. They have to know who is taking our data, for what purpose? Which protocol? I don’t want to position myself in that category of being weak. No, set your principles you get it? It is not a blanket sort of platform that we are all weak, maybe some not all (REC 14).

Those who thought it was a form of colonialism noted:

These research grants we are signing and which we are working under are crafted in a colonial way (Researcher 10).

And so, a grant comes in like that and you ask yourself is this ethical?. We have got the money; if you need the money, then you better agree that you are going to have un restricted access to the specimens and then the investigator is forced to put this in the consent form and because of lack of empowerment, it is like a false action. Very audience form of imperialism, it is not ethical (REC 2).

## Discussion

The extent and sustainability of data sharing in genomics research partly but highly depends on the ethicality of the practices in this process, whether real or perceived. In this study, trust and equity were identified as critical factors in genomic data sharing. Data sharing is not just a simple communication reveling of scientific information, but also a practical decision that is relational, embedded in social aspects of trust, equity, and technical capacities.

Our findings suggest that there are significant trust issues in collaborative genomic research. This finding is consistent with the literature from other African countries (13, 26). Due to past incidents of unethical research practices, there is a lack of trust in the use and sharing of research data and biological (27) samples. The findings align with a global survey that emphasized the importance of trust and how it influenced genomic data sharing (28). I agree with the importance of trust in the sharing of genomic data because the implications extend beyond individual participants to affect their families and society as a whole. What might be considered acceptable or ethical in one culture could conflict with deeply held values or privacy expectations in another.

While one would expect data sharing to come about through mutual dependence and equitable relationships, findings from our study suggest that genomic research collaborations tend to favor partners from the global North. Several researchers involved in genomic data sharing LMICs have pointed to unequal access to shared genomic data with most of it in the hands of collaborators from HICs which affects trust in collaborative research (26). Several of our participants except a few senior researchers raised concerns about inequitable access to shared data. This is a topic that has been occupying the thoughts of many people recently (9, 29), It’s worth exploring further to address these concerns to ensure that there is fair and equitable access to genomic data for everyone involved. These imbalances and power differentials have been attributed to the paucity of well-trained and knowledgeable African genomics scientists, the possibility of poor negotiation skills, and vulnerable positions including the preservation of colonial power relations between the global North and South (30, 31). The findings do not align with Mboowa et al., who indicated that the African continent has made significant progress with numerous aggressive sequencing campaigns led by African scientists, the development of infrastructure such as biobanks and biorepositories, and extensive training of emerging researchers in analytical skills by the H3Africa initiative (32).

Equitable access to data and possible benefit enhances trust in collaborative research (33–36). While researchers in LMICs may have limited access to the data shared with collaborators, it is crucial to determine who gets access and who is responsible for handling the data. Despite some of the developments mentioned above, the main difficulty faced by genomic researchers from LMICs is the scarcity of resources, inadequate infrastructure, a shortage of skilled personnel, and limited access to genomic tools for data manipulation and analysis (3, 37). There remains a need for further infrastructural development and capacity building to alleviate data access challenges. Research in this field may be viewed as fair or unfair depending on who is looking at it, thus the need for equity, fairness, and trust in collaborative research (7, 8, 30, 37, 38).

Researchers from LMICs have limited access to shared genomic data and possible benefits (18, 26), despite their significant contributions. Could this disparity be considered a lower moral status ascribed to LMIC researchers? (39). This disparity could mean that researchers from LMICs are being viewed as lesser individuals as compared to those from the Global North. This could be due to unequal power dynamics, historical exploitations, misuse of data, and a lack of regulatory frameworks to address the concerns of fairness and equity which affects trust in collaborative research (16, 40, 41).

Criticisms have been raised about the inequitable distribution of resources and research outputs (11). Limited research funding, inadequate training opportunities, and uneven benefit sharing have been identified as some of the challenges contributing to the imbalance between researchers in the global North and South (18). Researchers in the Global South often look out for collaboration opportunities but are frequently on the receiving end of such collaborations. Researchers in the Global South tend to trust that their northern partners have all the answers and agree to data-sharing arrangements spearheaded by their Northern counterparts. Power imbalances have been observed not only in collaborations between researchers from the Global North and South but also among researchers from the Global South (42). In some cases, academic researchers working in Global South institutions tend to assert ownership over participants and data. This raises the question of whether this power dynamic is unique to North-South collaborations or if it occurs more broadly among researchers from the Global South. The power struggles cut across and this could be attributed to the competitive academic environment, imbalances within institutions and the countries. It is essential to critically reflect on the existing power struggles between collaborating researchers, and how it affects genomic research in particular.

It could be a situation that some of the younger researchers from the South do not know the value of data as much as the collaborators from the North and are willing to share their data at the first opportunity creating a ground for exploitation. Reflections of the ethical concerns around the imbalances in the North-South collaborations which affect the trust and willingness of researchers from LMICs to share their genomic data are critical in preventing exploitation and maintaining scientific integrity for LMIC researchers (13, 43).

Data sharing can sometimes be perceived as a form of ethical imperialism (a situation where a code of ethical behavior or attitude is imposed on another society), particularly when inequities in access to data arise. Some of our participants considered data sharing a manifestation of ethical imperialism. This notion was attributed to unfavourable conditions, inequitable access to shared genomic data, and unfair sharing of the benefits of research. These findings concur with the literature, which shows that most collaborative agendas and policies are dominated by the Global North and are largely informed by the South’s dependency on the North (42, 44). Researchers from the Global South may not be aware of the potential benefits that may accrue from the shared data, and this may affect their bargaining power during benefit- and data-sharing negotiations. The issue arises when the communities that provided the genetic data, lack resources and yet they do not gain anything. The benefits of data sharing may also be affected by power imbalances caused by colonialism and Western policies that may not be well-suited to the African context (45). These unequal colonial relationships could be viewed as a form of ethical imperialism (3, 45, 46). Some of the more senior researchers in this study were not inclined to consider data sharing a form of ethical imperialism. Their thinking could be related to their personal experiences over the years.

It is important for researchers, institutions and RECs to be mindful of power dynamics in collaborative research and to strengthen contract review processes with legal support and capacity developments.

### Strengths

The main strength of this study was the involvement of various stakeholders in genomic research in Uganda. The findings provide an in-depth understanding of the value of trust and equity in genomic data sharing relevant to protecting research participants and researchers from data breaches, and inequality in collaborative research.

### Limitations

The main weakness of the study was the recruitment of researchers from one institution Makerere University College of Health Sciences which limits generalizability. However, the findings provide better understanding of a complex phenomenon of data sharing in collaborative genomic research. However, we strengthen the quality of the findings by engaging with REC members from different institutions across the country that approve genomics protocols and national regulators with experience in research oversight.

## Conclusion

Establishing relationships with international collaborators that are built on trust and equity are critical. Participants called for the development of a benefit sharing framework. Policymakers should consider developing regulatory frameworks that foster equity and fairness in data sharing and access in collaborative research. This will be facilitated by engaging in active advocacy dialogues with funders, and policymakers, and researchers from both the North and South. Researchers from the global South should have their capacities strengthened and empowered so they have the skills necessary to access and utilize the data as well as advocate favourably.

## Figures and Tables

**Table 1:** Summary of the methods

Setting	Sampling	Category	Sample size
MakCHS, IDI,UCI,Baylor Uganda,MAKBRC and IDRC	Purposive	Researchers	16
SBS,UCI,UVRI,MHREC,Lac or Hospital, JCRC and NARC	Purposive	REC members	14
UNCST,NDA,PDPO,UNHRO and Ministry of ICT	Purposive	Regulators	8

**Table 2:** Participant demographics of stakeholders involved in the study

Gender	Researchers	REC Members	Regulators
Male	12	10	5
Female	4	4	3
Total	16	14	8
Education level			
Diploma		02	
Bachelors	00	04	
Masters	6	06	07
PhD	10	02	1

**Table 3:** Emergent themes on (mis)trust and equity in collaborative genomic research

No.	Theme	Category
1.	(Mis)trust in collaborative research	• Fear of data misuse • Un clear motives for requiring data sharing • No benefit sharing framework • Commercialization • Perceptions held by researchers in the Global South about the North • Perceptions of the Global South by South
2.	Inequitable access to data	• Limited Infrastructure • Limited skillset • Concentration of data in the hands of a few powerful countries • Barriers to accessing data due to cost or lack of awareness)
3.	Power differentials between researchers and institutions in the Global North- South	• Unequal bargaining power • Culture of support in place (Yes, No) • Accountability
4.	Ethical imperialism and data sharing	• Data sharing considered a form of colonialism (Yes, No) • Value of the data shared? • Is it a weak mentality by researchers in LMICs?
